# Risk Factors for and Frequency of CT Scans, Steroid Use, and Repeat Visits in Inflammatory Bowel Disease Patients Seen at a Single-Center Emergency Department: A Retrospective Cohort Study

**DOI:** 10.3390/jcm10122679

**Published:** 2021-06-17

**Authors:** Lindsay Euers, Shaadi Abughazaleh, Kerri Glassner, Prianka Gajula, Michelle Jones-Pauley, Chika Ezeana, Mamta Puppala, Lin Wang, Stephen Wong, Ayah Oglat, Stephanie Nickerson, Bincy P. Abraham

**Affiliations:** Fondren IBD Program, Lynda K. and David M. Underwood Center for Digestive Disorders, Division of Gastroenterology, Hepatology, Houston Methodist Hospital, Weill Cornell Medical College, Houston, TX 77030, USA; sjabughazaleh@houstonmethodist.org (S.A.); klglassner@houstonmethodist.org (K.G.); pgajula@houstonmethodist.org (P.G.); mrjones-pauley@houstonmethodist.org (M.J.-P.); cfezeana@houstonmethodist.org (C.E.); mpuppala@houstonmethodist.org (M.P.); lwang3@houstonmethodist.org (L.W.); stwong@houstonmethodist.org (S.W.); oglataya@gmail.com (A.O.); snickerson.fnp@gmail.com (S.N.); bpabraham@houstonmethodist.org (B.P.A.)

**Keywords:** emergency medical services, inflammatory bowel disease, radiation, steroids, ulcerative colitis, Crohn’s disease

## Abstract

Patients with inflammatory bowel disease often present to the emergency department due to the chronic relapsing nature of the disease. Previous studies have shown younger patients to have an increased frequency of emergency department visits, resulting in repeated exposure to imaging studies and steroids, both of which are associated with risks. We performed a retrospective cohort analysis of inflammatory bowel disease patients seen at Houston Methodist Hospital’s emergency department from January 2014 to December 2017 using ICD codes to identify patients with Crohn’s disease, ulcerative colitis, or indeterminate colitis from the electronic medical record. Data were collected on demographics, medications, and imaging. Five hundred and fifty-nine patients were randomly selected for inclusion. Older age was associated with decreased risk of CT scan or steroid use. Patients with ulcerative colitis compared to Crohn’s had decreased risk of CT scan, while there was an increased risk of CT in patients on a biologic, immunomodulator, or when steroids were given. Steroid use was also more common in those with inflammatory bowel disease as the primary reason for the visit. Patients in our study frequently received steroids and had CT scans performed. The increased risk of CT in those on a biologic, immunomodulator, or steroids suggests more severe disease may contribute. Guidelines are needed to reduce any unnecessary corticosteroid use and limit repeat CT scans in young inflammatory bowel disease patients to decrease the risk of radiation-associated malignancy over their lifetime.

## 1. Introduction

Inflammatory bowel disease (IBD) is a chronic relapsing and remitting immune-mediated disease characterized by inflammation affecting primarily the gastrointestinal tract. There are two main subtypes of IBD, ulcerative colitis (UC) and Crohn’s disease (CD), which affect a significant portion of the United States population [1]. In 2014, it was reported that approximately 1.6 million Americans had IBD, with as many as 70,000 new cases diagnosed each year [2].

IBD has a substantial impact on the health care system and resource utilization. In fact, a recent study found that the health care costs of patients with IBD per year are over three times that of matched individuals without IBD [3]. The annual financial burden of IBD in the United States totaled greater than 31 billion dollars according to data collected between 1999 and 2005 [3,4,5]. Patients with IBD have high rates of emergency department (ED) use with an estimated 137,946 visits per year. Despite advances in therapy, IBD patients have had one of the largest increases in ED visits, constituting approximately 1% of all visits to the ED [6,7]. 

Previous studies evaluating ED visits in IBD patients have largely focused on demographic trends, predictors of inpatient admissions, and costs [8,9,10], with only a handful evaluating diagnostic or treatment modalities utilized during these visits [11,12,13,14,15,16]. Younger patients (aged 44 or less) have the highest rates of IBD-related ED visits [8,17,18]. Frequent ED visits lead to repeated CT scans. Based off data released from the Centers for Disease Control and Prevention (CDC) on adult patients presenting to the emergency department from 2007 to 2013, it is estimated that about 25–30% of patients with abdominal complaints undergo a CT scan annually [19]. The younger a patient is when a CT scan is performed, the higher the risk of malignancy over a lifetime [20,21,22,23,24], placing individuals with IBD at an increased risk of malignancy. Furthermore, corticosteroids are frequently given to IBD patients in the ED for acute disease exacerbations, either for those being admitted or to treat the patient temporarily while they are discharged to follow up with their gastroenterologist. Although steroids are often necessary to help induce remission, there are significant risks associated with steroids, especially with long-term use, and they should not be used for maintenance therapy. In certain cases, including intra-abdominal infections or abscesses related to IBD, or perianal fistulizing disease, corticosteroids are not useful and in fact can worsen outcomes [25,26]. 

Emergency department visits, steroids, and repetitive imaging in IBD patients account for significant costs, at times unnecessary utilization of resources, and are potentially harmful for patients over the course of their lifetime. The aim of our study was to examine the frequency and risk factors for CT scans, steroid use, and repeat ED visits in IBD patients seen at Houston Methodist Hospital’s ED over a 4-year period.

## 2. Materials and Methods

### 2.1. Design/Patient Population

A retrospective cohort study of IBD patients with at least one visit to Houston Methodist Hospital’s emergency department between 1 January 2014 and 31 December 2017 was performed. To identify patients from the electronic medical records, ICD codes were used for: ulcerative colitis, Crohn’s disease, or indeterminate colitis. Charts were reviewed individually to confirm IBD diagnosis, and those without IBD were excluded. 

### 2.2. Data Collection

Data were collected on demographics (age, sex, and ethnicity); medications (biologics, immunomodulator, and steroids); and imaging (CT abdomen/pelvis). The number of times a patient visited the ED during the study period was documented. The reason for the ED visit and whether or not steroids were given specifically for IBD were included. The location that the CT scan was performed at (ED or after admission) was also documented. 

### 2.3. Statistical Analysis

Continuous variables were reported as mean (±standard deviation) or median (interquartile range) as appropriate and categorical variables as frequencies and proportions. All analyses were performed using IBM SPSS Statistics Version 25.0 (IBM Corp, Armonk, NY, USA). A linear regression model was used to analyze the impact of risk factors on the dependent variables, the coefficients were given, and a *p*-value of <0.05 was considered to indicate statistical significance in the impact of the risk factor on the dependent variable.

### 2.4. Ethical Considerations

The study was conducted in compliance with the Health Insurance Portability and Accountability Act and institutional review board approval was obtained. 

## 3. Results

### 3.1. Patient Characteristics

**Figure 1:**
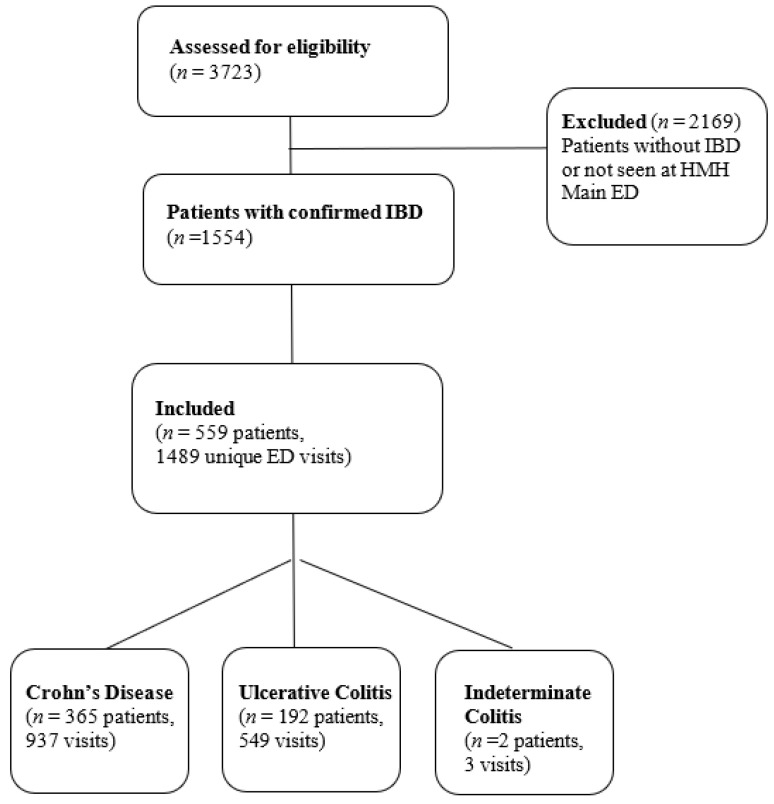
Consort diagram: frequency of visits, CT scans, and steroid use in inflammatory bowel disease patients seen in the emergency department: a retrospective cohort study.

There were 3723 patients identified by ICD codes. After individual chart review, the diagnosis of IBD was confirmed in 1554 patients. Among these, 559 unique patients were randomly selected for inclusion in the study. These patients had a total of 1489 unique ED visits (Figure 1).

**Figure 2:**
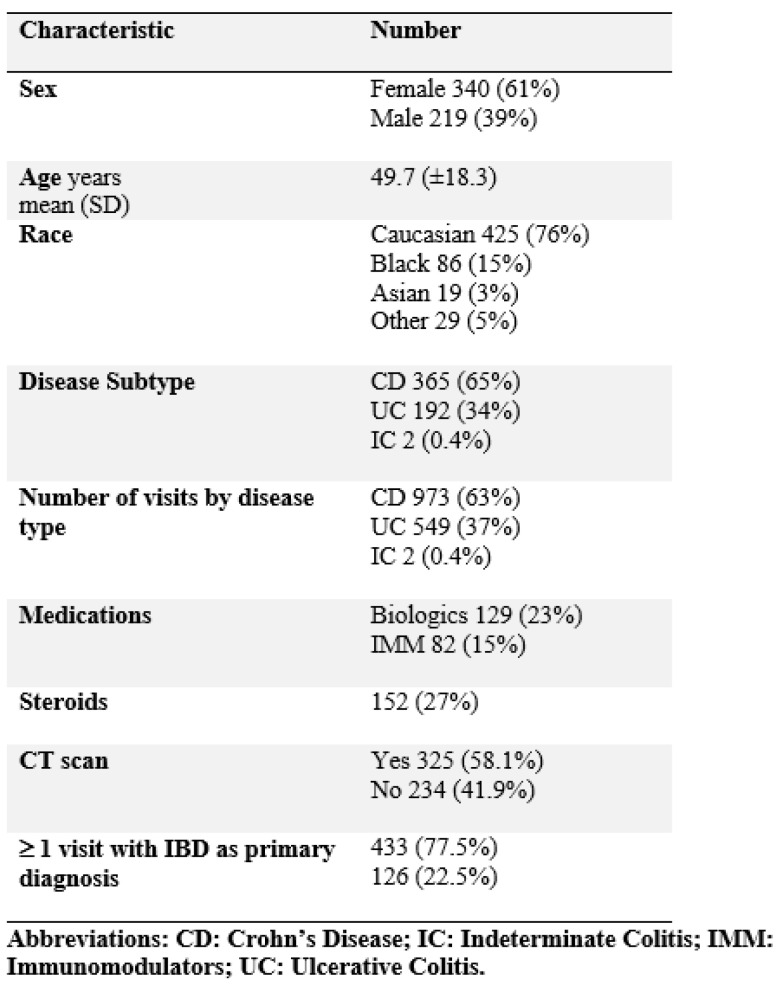
Baseline patient characteristics.

Of the 559 patients, 340 (61%) were female and 219 (39%) were male. The mean age was 49.7 years (± 18.3) (range 18–95). Caucasians, with a total of 425 patients (76%), were the majority, while 86 (15%) were Black, 19 (3%) were Asian, and the rest were of other races. Crohn’s disease (CD) accounted for 365 (65%) of the patients and 937 (63%) of the total visits. Ulcerative colitis (UC) accounted for 192 (34%) of the patients and 549 (37%) of the total visits. Indeterminate colitis accounted for 2 (0.4%) of the patients and 3 (0.2%) of the total visits. Biologic medications were used in 129 (23%) patients, including infliximab, adalimumab, vedolizumab, certolizumab, ustekinumab, and golimumab. Immunomodulators were used in 82 (15%) including azathioprine, 6-mercaptopurine, and methotrexate. There were 152 (27%) treated with steroids specifically for inflammatory bowel disease. A total of 433 (77%) of patients had at least one ED visit with IBD as the primary reason for the visit. There were 325 patients (58%) who had a CT scan performed (Figure 2). 

### 3.2. Repeated Visits to the Emergency Department

**Figure 3:**
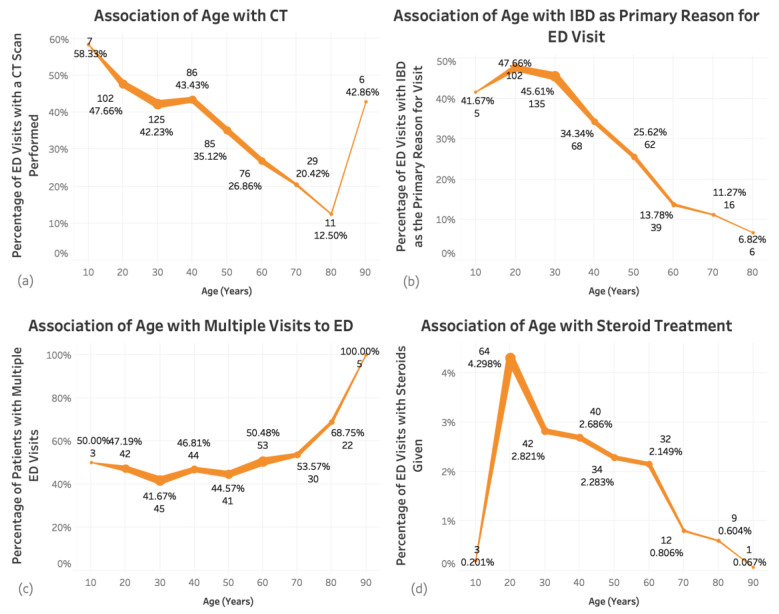
Association of age with: CT scan (**a**), IBD as primary reason for visit (**b**), multiple ED visits (**c**), and steroid treatment (**d**). Percentage of visits out of the total number of visits for each age grouping is shown on the x-axis. Line thickness indicates the number of visits, with thicker lines representing more visits. Older age was associated with decreased risk of a CT scan (*p* ≤ 2.2 × 10^−16^, coefficient −0.002), decreased risk of steroid use (*p* = 4.039 × 10^−9^, coefficient −0.0008), and increased likelihood of having multiple ED visits (*p* = 0.005, coefficient 0.001).

There were 257 patients (46%) who had multiple ED visits for a total of 1187 unique visits. The average age of those with multiple ED visits was 51.8 (± 19.6) years, 160 (62%) were women, and 160 (62%) had CD. Among this group, 58 (23%) were treated with a biologic and 44 (17%) with an immunomodulator. IBD was the primary reason for the ED visits in 312 (26%) of the 1187 visits. Thus, 130 patients (51%) had at least one ED visit primarily for IBD. In addition, steroids were given specifically for IBD in 82 (32%) patients, and 168 (65%) had a CT scan performed during at least one ED visit (Figure S2). There was an increased likelihood of having multiple ED visits with older age (*p* = 0.005, coefficient 0.001) (Figure 3). 

**Figure 4:**
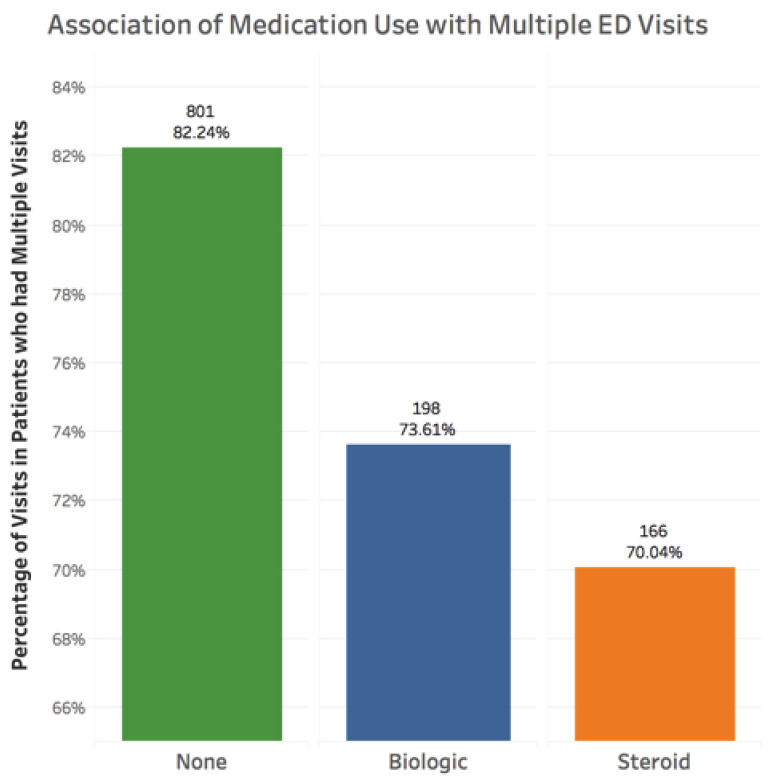
Association of medication use with visits in patients with multiple ED visits. There was a decreased likelihood of multiple ED visits with biologic use (*p* = 0.023, coefficient −0.055) or if patients were previously taking steroids for IBD (*p* = 0.0005, coefficient −0.083).

**Figure 5:**
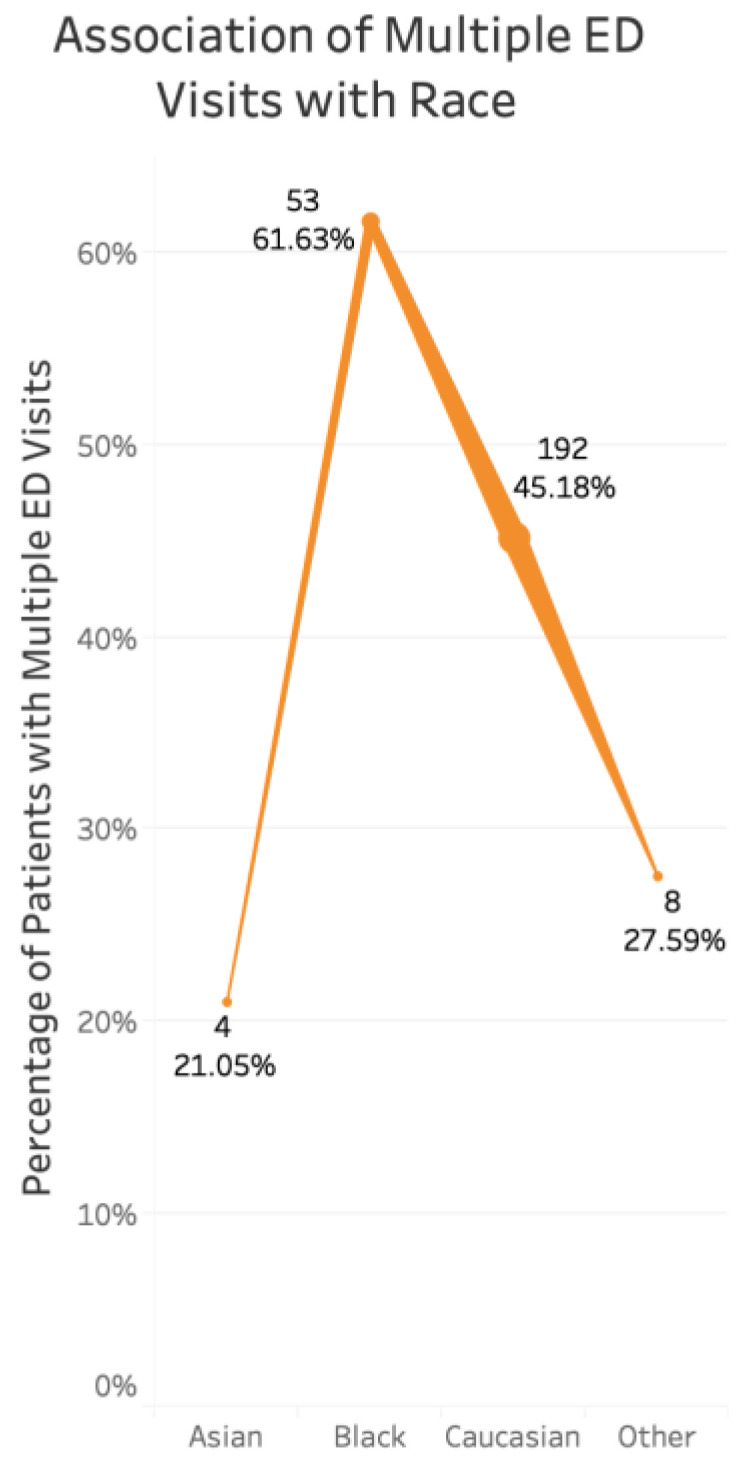
Association of multiple ED visits with race. There was a significant association between race and multiple visits, with Blacks (*p* = 1.69 × 10^−8^, coefficient 0.439) and Caucasians (*p* = 3.66 × 10^−5^, coefficient 0.310) comprising the majority of these visits. Line thickness indicates the number of visits, with thicker lines representing more total visits.

There was a decreased likelihood of multiple ED visits with biologic use (*p* = 0.023, coefficient −0.055), if patients were previously taking steroids for IBD (*p* = 0.0005, coefficient −0.083) (Figure 4), and among those with IBD as the primary reason for the visit (*p* = 0.014, coefficient −0.059). There was a significant association between race and multiple visits, with Blacks (*p* = 1.69 × 10^−8^, coefficient 0.439) and Caucasians (*p* = 3.66 × 10^−5^, coefficient 0.310) comprising the majority of these visits (Figure 5). There was no association found with sex or immunomodulator use. 

### 3.3. CT Scans

**Figure 6:**
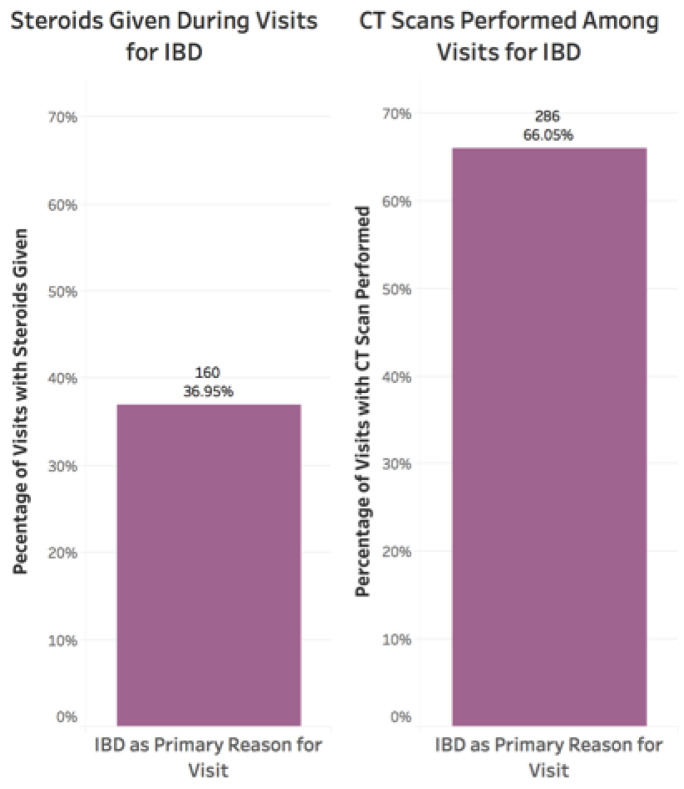
Percentage of visits with steroids given or CT scans performed in visits with IBD as the primary reason for the visit. Steroid use was also more common in those with IBD as the primary reason for the ED visit (*p* ≤ 2.2 × 10^−16^, coefficient 0.228). There was an increased risk of CT scan when the primary reason for the visit was for IBD (*p* ≤ 2.2 × 10^−16^, coefficient 0.372).

Among the 325 (58%) patients who had a CT scan performed, there were 599 unique visits to the ED. The CT scan was ordered in the ED in 527 (88%) of the visits. The mean age of patients who had a CT scan was 47 (± 17.8), 194 (60%) were women, and 223 (69%) had Crohn’s disease. IBD was the primary reason for the visit to the emergency department in 286 (48%) of visits. Among this group, 77 (24%) were on a biologic medication, 54 (17%) on an immunomodulator, and 125 (39%) received steroids (Figure S3). Older age was associated with a decreased risk of a CT scan (*p* ≤ 2.2 × 10^−16^, coefficient −0.002) (Figure 3), as well as UC compared to CD (*p* = 0.009, coefficient −0.063) (Figure S1). There was an increased risk of CT scan in patients on a biologic (*p* = 0.04, coefficient 0.04), immunomodulator (*p* = 0.04, coefficient 0.03), and when steroids were given specifically for IBD (*p* = 1.44 × 10^−13^, coefficient 0.102) (Figure S2). There was an increased risk of CT scan when the primary reason for the visit was for IBD (*p* ≤ 2.2 × 10^−16^, coefficient 0.372) (Figure 6).

### 3.4. Steroid Utilization

There were 167 (30%) patients who received steroids for IBD during 262 unique visits (22%). Among this group of patients receiving steroids for IBD, the mean age was 46 (±18), 95 (57%) were female, and 115 (69%) had Crohn’s disease. There were 52 (31%) on a biologic medication and 38 (23%) on an immunomodulator (Figure S4). Increasing age was associated with decreased steroid use (*p* = 4.039 × 10^−9^, coefficient −0.0008) (Figure 3). Males were more likely to be given steroids (*p* = 0.005, coefficient 0.048) (Figure S3), as were those on a biologic (*p* = 0.001, coefficient 0.059) or immunomodulator (*p* = 2.617 × 10^−16^, coefficient 0.131) (Figure S4). Steroid use was also more common in those with IBD as the primary reason for the ED visit (*p* ≤ 2.2 × 10^−16^, coefficient 0.228) (Figure 6).

## 4. Discussion

In this retrospective cohort study, we evaluate the characteristics of IBD patients seen at a single-center ED, and the relationship these characteristics have on important outcomes including repeat ED visits, steroid use, and CT scans. Repeat ED visits occurred in approximately half of the IBD patients studied. However, multiple ED visits were more common when the primary reason for the visit was not IBD. There was also an increased likelihood of multiple ED visits in patients who were older. Older patients are more likely to have medical comorbidities other than IBD which might necessitate an ED visit. Furthermore, Black, and Caucasian patients were more likely to have multiple ED visits. It has previously been shown that Black IBD patients were less likely to be under the care of a gastroenterologist or IBD specialist and are more likely to visit the ED, likely related to barriers to health care access [27]. Importantly, we found a decrease in the likelihood of repeated ED visits in patients who were being treated with biologics for IBD, indicating that appropriate IBD treatment may reduce the risk of repetitive visits and potentially translate to appreciable cost-savings. Consistent with this, Rahman et al. found that rates of hospitalizations, ED visits, and inpatient surgeries markedly declined for Crohn’s patients in Ontario from 2003 to 2014, while rates of biologic medication use increased [28]. This information is reassuring; repeat ED visits are less likely to be for IBD and appropriate IBD treatment seems to play a role in preventing repeat visits. 

We found that CT scans were performed in IBD patients quite frequently, with the majority occurring in the ED rather than after admission. In our study, 69% of patients with CD and 31% of those with UC were subjected to imaging. Another retrospective study of almost 650 patients with CD in two emergency departments found similar results to ours [16]. Consistent with previous studies, we found higher rates of imaging use in patients with CD compared to UC [12] and in younger patients [29]. 

A typical CT abdomen and pelvis equates to approximately 10 millisievert (mSv) of radiation. It has been estimated that approximately 29,000 future cancer diagnoses could be related to CT scans performed in the US in 2007 alone [30]. Desmond et al. evaluated 354 patients with Crohn’s disease over a mean follow up period of 6.7 years and found increasing use of imaging studies over the time period with a mean effective dose of radiation of 36.1 mSv. Of the 354 patients evaluated, 55 of the patients exceeded 75 mSv (effective dose equivalent to 3750 standard chest X-rays). In patients under the age of 45, a dose response to radiation has been observed to be associated with thyroid cancer, leukemia, and non-Hodgkin lymphoma [31]. Exposure to diagnostic radiation has been shown to be higher among patients with clinical markers of increased disease severity and patients who required 6-mercaptopurine, azathioprine, or infliximab [15]. Likewise, we found patients on a biologic or immunomodulator and those in whom the primary reason for the ED visit was IBD to be at increased risk of CT scan. One explanation for this is that patients who require more aggressive medical therapy may have more severe disease prompting imaging.

While imaging may aid the emergency physician in diagnostic evaluation of a patient with IBD, guidelines are needed to not only limit repeat CT scans, but also to ensure that the imaging modalities selected provide information that guide care. This might include scoring tools such as demographic, clinical, and laboratory data for risk stratification, use of alternative imaging modalities (MRE, MRI, low-dose CT), or in the appropriate situation, foregoing imaging and proceeding with direct visualization through endoscopy [32]. Examples may be as simple as instructing practitioners to obtain an MRE for patients under the age of 50 and a CTE in patients over the age of 50 when evaluating small bowel disease. Retrospective studies have attempted to use various predictors to identify patients with IBD presenting to the ED in whom a CT leads to clinically actionable findings. These studies seem promising; however, no clinical decision rule has been validated to identify patients in whom imaging can be avoided or delayed [11,12,13,14]. For now, we hope the data obtained in our study can be used to educate our ED physicians about the frequency and risk of CT scans in the younger IBD population seen at our hospital.

Steroids have long been a primary medical therapy for induction of remission in IBD [33,34,35,36]. Patients exposed to repeated courses or an extended duration of steroids are at risk for multiple side effects including hypertension, peptic ulcers, weight gain, impaired glucose tolerance, accelerated bone mineral loss, cataracts, and accelerated atherosclerosis among others [37]. In our study, we found steroid use during ED and hospital admissions to be relatively common despite the increasing availability of alternative medications for IBD. Interestingly, steroid use was associated with biologic and immunomodulator use, suggesting that the ongoing utilization tends to occur in patients with more severe disease.

There were several limitations to our study. First, the study was conducted in an academic tertiary referral center and might not be applicable to other ED settings. Patients in this setting often have increased complexity compared to those seen in the community ED. Furthermore, the number of ED visits for each patient was likely underestimated as only visits to our ED were included in the study. Finally, this was a retrospective study and therefore subject to confounding. 

## 5. Conclusions

Our study of IBD patients presenting to a single-center ED evaluated the frequency of and risk factors for CT scans, repeated ED visits, and steroid administration. Repeat ED visits occur frequently and place young IBD patients at risk of having multiple CT scans with radiation exposure that can be costly and potentially harmful. Similarly, the use of steroids continues to be common despite alternative medical therapies and the known side effects. By better understanding the characteristics associated with repeat ED visits, CT scans, and steroid use in patients with IBD, emergency department physicians and gastroenterologists can work together to optimize resource utilization and improve patient care.

## Data Availability

The data presented in this study are available on request from the corresponding author. The data are not publicly available due to HIPPA.

## Supplementary Materials

The following are available online at https://www.mdpi.com/article/10.3390/jcm10122679/s1: Figure S1. Association of CT scan with IBD subtype. There was a decreased risk of CT scan with ulcerative colitis compared to Crohn’s disease (*p* = 0.009, coefficient −0.063); Figure S2. Association of CT scan with medication use. Medications that patients were on (biologic, immunomodulator, steroids, or none) are shown on the x-axis with percentage of ED visits on the y-axis. There was an increased risk of CT scan in patients on a biologic (*p* = 0.04, coefficient 0.04), immunomodulator (*p* = 0.04, coefficient 0.03), and when steroids were given for IBD (*p* = 1.44 × 10^−13^, coefficient 0.102); Figure S3. Association of steroid use with gender. Males were significantly more likely to be given steroids compared to females (*p* = 0.005, coefficient 0.048); Figure S4. Medications used in IBD patients given Steroids. Patients on a biologic (*p* = 0.001, coefficient 0.059) or immunomodulator (*p* = 2.617 × 10^−16^, coefficient 0.131) were more likely to be given steroids compared to patients not on either of these medications.
