# Supplementary material for: Risk Factors for and Frequency of CT Scans, Steroid Use, and Repeat Visits in Inflammatory Bowel Disease Patients Seen at a Single-Center Emergency Department: A Retrospective Cohort Study
Source: J Clin Med. 2021 Jun 17;10(12):2679. doi: 10.3390/jcm10122679 (PMC8234275; doi:10.3390/jcm10122679)
Supplement: Supplementary file 1 [file jcm-10-02679-s001.zip › jcm-1221009-supplementary.pdf]

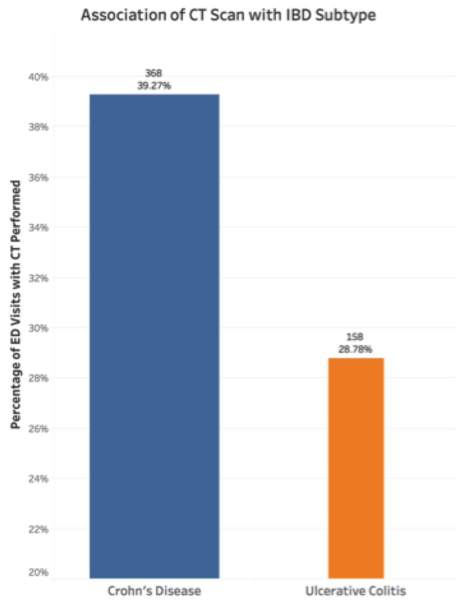

Figure S1. Association of CT scan with IBD subtype. There was a decreased risk of CT scan with ulcerative colitis compared to Crohn's disease ($p = 0.009$, coefficient −0.063).

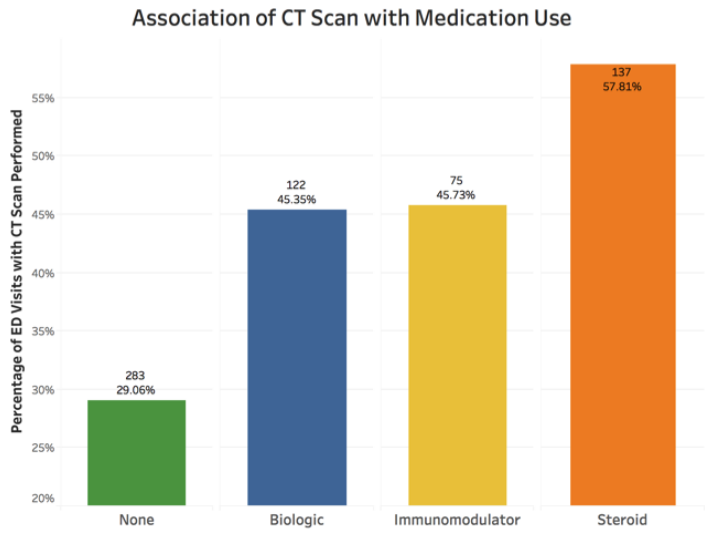

Figure S2. Association of CT scan with medication use. Medications that patients were on (biologic, immunomodulator, steroids, or none) are shown on the x-axis with percentage of ED visits on the y-axis. There was an increased risk of CT scan in patients on a biologic ($p = 0.04$, coefficient 0.04), immunomodulator ($p = 0.04$, coefficient 0.03), and when steroids were given for IBD ($p = 1.44 \times 10^{-13}$, coefficient 0.102).

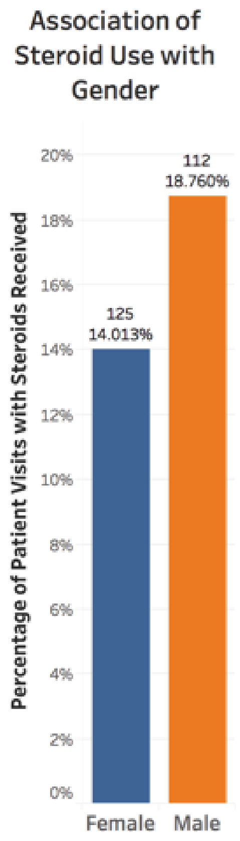

Figure S3. Association of steroid use with gender. Males were significantly more likely to be given steroids compared to females ($p = 0.005$, coefficient 0.048).

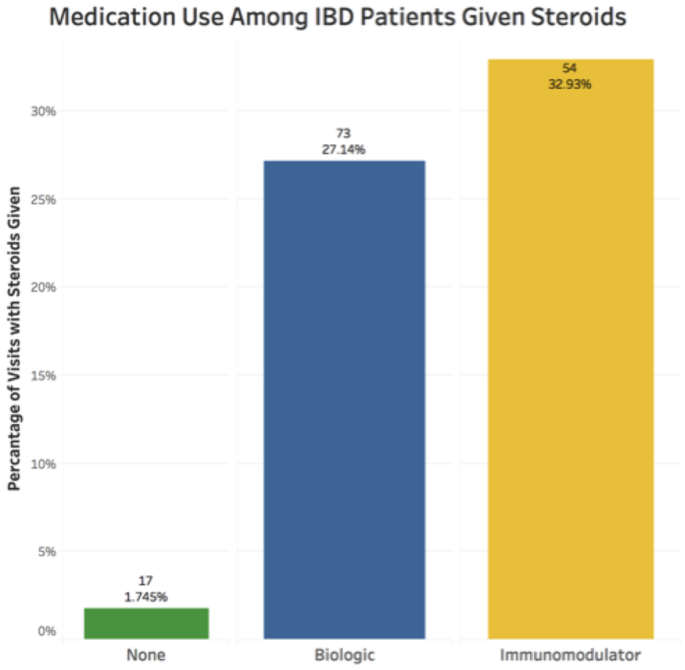

Figure S4. Medications used in IBD patients given Steroids. Patients on a biologic ($p = 0.001$, coefficient 0.059) or immunomodulator ($p = 2.617 \times 10^{-16}$, coefficient 0.131) were more likely to be given steroids compared to patients not on either of these medications.
